# Lessons I have learned from my patients: everyday life with primary orthostatic tremor

**DOI:** 10.1186/s40734-016-0048-5

**Published:** 2017-01-12

**Authors:** Marie Vidailhet, Emmanuel Roze, Lucie Maugest, Cécile Gallea

**Affiliations:** 1 Institut National de la Santé et de la Recherche Médicale (INSERM), U 1127, F-75013 Paris, France; 2 Centre National de la Recherche Scientifique (CNRS), UMR 7225, F-75013 Paris, France; 3 Sorbonne Universités, UPMC University Paris 06, UMR S 1127, F-75013 Paris, France; 4 Institut du Cerveau et de la Moelle épinière, ICM, F-75013 Paris, France; 5 Assistance Publique Hôpitaux de Paris (APHP), Département des Maladies du Système Nerveux, Hôpital Pitié-Salpêtrière, F-75013 Paris, France; 6 Department of Neurology, University of Dijon, Dijon, France; 7 Centre de Neuroimagerie de Recherche (CENIR), Institut du Cerveau et de la Moelle, ICM, F-75013 Paris, France; 8 Department of Neurology, Salpetriere Hospital, Bd de l’Hopital, 75013 Paris, France

## Abstract

**Background:**

Primary orthostatic tremor is a rare disorder that is still under-diagnosed or misdiagnosed. Motor symptoms are fairly characteristics but the real impact on the patient’s every day life and quality of life is under-estimated. The ”how my patients taught me” format describes the impact on the patients’ every day life with their own words, which is rarely done.

**Case presentation:**

A 46 year old lady was diagnosed primary orthostatic tremor (POT) based on the cardinal symptoms: feelings of instability, leg tremor and fear of falling in the standing position, improvement with walking and disappearance while sitting, frequency of Tremor in the 13–18Hz range, normal neurological examination. She gives illustrative examples of her disability in every day life activity (shower, public transportation, shopping). She reports how she felt stigmatized by her “invisible disorder”. As a consequence, she developed anxiety depression and social phobia. All these troubles are unknown or under recognized by doctors and family.

**Conclusions:**

We review the clinical signs of POT that may help to increase the awareness of doctors and improve the diagnosis accuracy, based on the motor symptoms and description of the every day life disability, as reported by the patient. Non-motor symptoms (including somatic concerns, anxiety, depression, and social phobia) should be better considered in POT as they have a major impact on quality of life. Pharmacological treatments (clonazepam, gabapentin) may be helpful but have a limited effect over the years as the patients experience a worsening of their condition. On the long term follow-up, there are still unmet needs in POT, and new therapeutic avenues may be based on the pathophysiology by modulating the cerebello-thalamo-cortical network.

**Electronic supplementary material:**

The online version of this article (doi:10.1186/s40734-016-0048-5) contains supplementary material, which is available to authorized users.

## Background

Feelings of instability and fear of falling are frequent complaints in elderly subjects and have a strong impact on quality of life. Although these features are fairly typical of primary orthostatic tremor (POT) [1], this rare condition is likely under-diagnosed (mean delay of diagnosis = 4.5–9.6 years, range = 0–44) [2–4].

Nevertheless, this condition is relatively easy to diagnose when you listen carefully to your patients, as they often report the main clinical features: lower body tremor activated upon standing (with feelings of unsteadiness and decreased time immobile in the upright position) which is improved by walking and absent when sitting or lying down. The unique electrophysiological signature characteristic of primary orthostatic tremor is a 13–18 Hz tremor in the lower limb muscles (and sometimes the trunk) in the standing position [1–4].

## Case presentation

A 46-year-old lady was diagnosed with “primary orthostatic tremor” after 10 years of undiagnosed symptoms: she complained of fatigue, stiffness of the legs, fear of falling and feelings of instability. Her first symptoms occurred when she had to stand still for an unusually long time (she was a singer in an amateur choir). Over time, three general practitioners examined her, and the proposed diagnoses were depression, chronic fatigue syndrome, and functional disorders, for which she was prescribed antidepressant therapy without any beneficial effect. Eventually, based on the description of her clinical symptoms, a neurologist suspected the diagnosis of POT when she mentioned that the symptoms occurred in the standing position. Neurological examination was normal, however, in a prolonged standing position, the neurologist observed fast trembling of the hem of her skirt, revealing her leg and thigh orthostatic tremor. Electrophysiological assessment confirmed the diagnosis of POT (frequency: 17Hz) Gabapentin (up to 300 mg/day) was not well tolerated. Clonazepam (3 mg/day) was prescribed with a partial benefit (initial subjective improvement of 50% in daily life) and was increased up to 4 mg/day according to the patient’s needs.

Although the patient further increased the dose of clonazepam up to 4.5 mg/day (higher doses were not tolerated due to drowsiness), she reported that the duration of standing position without support decreased progressively over the following 5 years, with an important impact on her every day life. Here is the description of everyday life with POT by the patient: “Each morning presented numerous obstacles: I have to sit to take a shower and wash my hair; I could not use the hairdryer standing in front of the mirror and I hastily brush my teeth and put on her makeup; I have to put on my trousers while seated. Cooking breakfast was not without its challenges either: I constantly have to lean on the surroundings while washing the dishes, and I am afraid to use a stool to put away her dishes in a high cupboard. Then, I race to the metro. One particular morning, I felt very uncomfortable standing in a compact crowd and asked a fellow passenger for a seat. The passenger looked very annoyed and muttered “you are not disabled!”, and I felt ashamed. My work environment is also full of uncomfortable moments in which standing is hard to avoid, such as taking the elevator, making copies at the copy machine, and long lines in the cafeteria. I would deliberately avoid friends to skip long conversations in the standing position, which elicited quizzical looks: “maybe she isn’t in a good mood?”, they would think. When I went back to my desk, I felt exhausted although it was only mid-day. In the evening, shopping at the grocery was a nightmare, especially standing in line at the cashier. I would skip chorale rehearsal, as it was too tiring to remain standing. Moreover, my family did not understand why I was so sad, depressed, anxious, and “phobic” about many activities, such as going out, visiting exhibitions, etc. I feel stigmatized by an invisible disease, and I have progressively lost my self-esteem; I had the impression of being misunderstood and that people were not taking my troubles seriously. The diagnosis was a relief. At last, I could explain my troubles in simple words. Eventually, I have joined the patient’s association in order to increase the awareness of people and doctors.

## Discussion and conclusions

What I have learned from this patient and many others is that the impact on daily life is under-estimated, as doctors often do not realize how many activities may become restricted or impossible with POT. As illustrated by this observation, the detailed description of limitations in everyday life activities, with special attention to those while standing, corresponds to the (mainly motor) key diagnosis features [1–4] of POT (Table 1) and may help non-neurologists to be aware of the diagnosis despite its rarity. An additional clinically relevant clue upon neurological examination may be the “hem sign”, fast trembling of the hem of the skirt or long shirt covering the thigh of the patient, reflecting the high frequency leg tremor in the prolonged standing condition (Additional file 1: Video S1). High frequency (13–18 Hz) rhythmic bursts of muscular activity can be detected on surface electromyography in POT, as part of the diagnosis criteria established by the Movement Disorders Society [1]. This patient also reports the lesser well-known non-motor symptoms, such as anxiety, depression, social avoidance and reduction of leisure activities, loss of self-esteem, fatigue, stigma, and self-withdrawal. This patient’s emotional experience is not unique. Many of our POT patients had anxiety as part of the emotional burden [3]. Moreover, fatigue, depression and social avoidance reported our patient are in line with the higher scores for somatic concerns, anxiety related disorders, depression and antisocial features described in a recent case control study including 16 POT patients [5, 6].

**Table 1:** Characteristics of primary orthostatic tremor

Motor features	Non motor features	Electrophysiological & neuro-imaging findings
Lower body tremor activated on standing position	Fear of falling	Tremor frequency 13–18 Hz (high inter-muscular coherence)
Tremor absent when sitting and lying	Pain	Brain neuroimaging (MRI) normal range
Primarily affects the legs and trunk (Tremor may be observed in the shoulders/arms while the patient is presses the hands on a table to support himself/herself)	Anxiety Depression	Normal DAT scan Cerebello-thalamocortical structural and functional abnormalities
Unsteadiness while standing	Social phobia (Self-withdrawal)	
Urge to search support to feel stable		
Worsening over time (same tremor frequency, increased amplitude) Arm postural tremor (6–8Hz)	Alteration of attention, executive function, visuospatial ability, & visual memory (>60 y.o.)	

*MRI* magnetic resonance imaging, *DAT scan* dopamine transporter imaging, *y.o.* years-old



**Additional file 1: Video S1.** The “hem sign”: fast trembling of the hem of the skirt, revealing legs and thighs orthostatic tremor. (MP4 507 kb)


Overall, the recent cases series on large number of patients [2–4] report that the majority of POT cases were in women (63 to 76%) with a wide range of age at onset (range 37–88) [3, 4] with similar clinical descriptions of symptoms and consistent high-frequency tremor, pathognomonic of this disorder. The outcome of the condition was poor. On long-term follow-up (5–25 years) [3], there was not change of tremor frequency but 80 to 90% of the patients reported that their symptoms became more severe, with immediate instability upon standing, and falls in 24% of cases [4]. Arm tremor was observed on leaning and, over time, 70% of the patients developed postural upper limb tremor [3]. The patients who reported symptom worsening had a longer duration (median 15 years) [3]. Treatments were overall unsatisfactory over time. Clonazepam appears to be the most effective (one third of the patients with moderate or marked improvement) followed by gabapentin (900–1800 mg/day) [2–4]. The effects of other medications were even more limited and inconsistent (Table 2). The beneficial effect of clonazepam diminished over time and few patients experienced benefit from subsequent drugs [4]. Reports of thalamic deep brain stimulation and spinal cord stimulation are scarce. Recent advances in neuroimaging have pointed towards the cerebello-thalamo-cortical network [7]. This paves the way to network-based innovative treatments, as medical and surgical interventions are only partially efficient [3, 4, 8].

**Table 2:** Treatment options in primary orthostatic tremor

Drugs/Neurostimulation	Doses	Reported clinical effect
Clonazepam	0.5 mg- 6 mg /day	Moderate to marked benefit in 50 to 30% of the patients
Gabapentin	300–2400 mg/day	Moderate to marked benefit
Beta-blockers (propranolol	20–240 mg/day	Little effect of POT. May improve arm postural tremor
Primidone	125–250 mg/day	No effect, poor tolerance
L-Dopa Pramipexole^a^	300–800 mg/day	Rare cases (short term benefit)^b^
Antiepileptic drugs (valproic acid, phenobarbital, carbamazepine, levetiracetam, topiramate, pregabalin)		Minimal to no effect Few cases. No prolonged treatments
^c^Deep Brain stimulation Spinal cord stimulation	Thalamus^c^	Rare cases. Variable results. Some increase in time in the upright position.
Botulinum toxin (tibial anterior)		No beneficial effect

*PD* Parkinson’s disease

^a^Anecdotal effect of pramipexole ^b^L-Dopa or pramipexole may help in slow orthostatic tremor in Parkinson’s disease, little or no benefit on POT preceding or associated with PD. ^c^Deep brain stimulation of the thalamus (Ventral intermediate nucleus Vim) same target as in Essential Tremor

POT mostly appears as an isolated disorder as up to 85% of the cases never evolved towards another condition [3, 4]. Those with additional disorders were named “orthostatic tremor plus” [9] and were mainly associated with parkinsonism, restless legs syndrome, tardive dyskinesia or mild cognitive impairment. Some patients with unsteadiness upon standing may be mistaken for POT but are found to have slow orthostatic tremor on neurophysiological testing. They have greater gait disturbances at an early stage and a higher risk of falls [10] and overall, slow orthostatic tremor (<10Hz) bears little resemblance with POT [10]. Extensive description of secondary orthostatic tremors is beyond our scope as a number of them did not have the typical high frequency tremor [11, 12]. Some findings are in favour of secondary orthostatic tremor (Table 3). Medic, Dysmetria, ataxia, eye movement disorders, sensory deficits or limbs weakness, combined with medical history (including neuroleptics of recreational solvants absorption) and structural abnormalities on brain or spinal cord imaging are valuable clues for symptomatic orthostatic tremor (Table 3) [11, 12].

**Table 3:** Secondary orthostatic tremors

Associated clinical features neuro-imaging abnormalities	High/slowtremor frequency	Neurological disorders
Parkinsonism, gait difficulty, postural instability	Slow 6–7 Hz tremor	Acqueduc stenosis
Truncal ataxia, cranial nerve involvement	Fast orthostatic tremor (15 Hz)	Pontine lesions/midbrain lesions
Broad based ataxic gait, cerebellar tremor, dysmetria, speech involvement, saccadic pursuit, dysmetria of saccades	From fast 14–15 Hz tremor to the lower range of OT (13 Hz tremor)	Cerebellar degeneration, Spino-cerebellar ataxia (genetic, e.g. SCA2)
Ataxia, sensory disturbances, pyramidal signs, relapsing remitting/progressive	Very slow 4 Hz tremor	Multiple sclerosis
Postural instability, urinary symptoms	14–13 Hz	Spinal cord lesion
Sensory disturbances, mild weakness of the upper limbs, postural tremor	6–7 Hz	Neuropathy (IgG and IgA gammapathy or polyradiculopathy, paraneoplasic disorders

Rare cases of orthostatic tremor have been reported with dopamine blocker agents (neuroleptics), vitamin B12 deficiency (same frequency as POT°, spastic paraparesis (16 Hz), stiff-person and Graves disease

Overall, careful clinical history and clinical examination, may helpneurologists and non neurologists to detect patients with POT, who were misdiagnosed and did not receive adequate treatments and attention.
